# Myxoid Pleomorphic Liposarcoma of the Spermatic Cord: A Rare Entity at a Rare Site

**DOI:** 10.7759/cureus.65554

**Published:** 2024-07-28

**Authors:** Yazhini Chandrasekaran, Kalaivani Amitkumar, Ashwini Elamaran, Muthu Sudalaimuthu, Subhalakshmi Kumaran

**Affiliations:** 1 Department of Pathology, Sri Ramaswamy Memorial (SRM) Institute of Science and Technology, Chengalpattu, IND; 2 Department of Radiology, Sri Ramaswamy Memorial (SRM) Institute of Science and Technology, Chengalpattu, IND

**Keywords:** adipocytic tumors, liposarcoma of spermatic cord, myxoid pleomorphic liposarcoma, paratesticular tumors, liposarcoma

## Abstract

Myxoid pleomorphic liposarcoma (MPLPS) is an extremely rare entity that has been recognized and included in the literature recently. Liposarcomas are adipocytic tumors that are usually located in the extremities and retroperitoneum. Paratesticular liposarcomas are extremely rare malignant tumors originating from the adipose tissue of the paratesticular region. Myxoid pleomorphic liposarcoma (MPLPS) is an exceedingly rare variant of liposarcoma, with very few cases reported in the literature so far. Mediastinum is the most common site for MPLPS followed by the limbs, head, and neck. We report a case of a 50-year-old male patient who presented with a swelling in the right inguinal region, which came to the patient's attention in the past month. After investigations, a right-high orchidectomy was done. Histopathological examination and immunohistochemistry were performed and a diagnosis of myxoid pleomorphic liposarcoma (MPLPS) involving the spermatic cord was made. So here we report this case of myxoid pleomorphic liposarcoma involving the spermatic cord for the first time in the literature.

## Introduction

The paratesticular area, which comprises the spermatic cord, testicular tunics, epididymis, and vestigial remains, is where paratesticular malignancies originate. The spermatic cord is the most often affected site [1]. These tumors can arise by malignant transformation of a pre-existing lipoma or can also originate de novo from the tissue of the cord, which can be an extension of retroperitoneal fat [1]. This tumor mostly presents as a growing painless mass in the inguinal or scrotal region. Liposarcoma of the spermatic cord itself is a very rare entity, and only less than 200 cases have been reported in the literature [2]. These tumors are commonly misdiagnosed without histopathology which leads to local recurrence and improper treatment [3]. Myxoid pleomorphic liposarcoma (MPLPS) is a newly described entity and was incorporated by WHO in 2020. MPLPS are extremely rare tumors, aggressive in nature, with a high recurrence rate, and they metastasize to the lungs, bone, and soft tissues at an earlier stage [4]. With the available literature review, it was found that this rare tumor has a predilection towards mediastinum in most cases but may also involve limbs, head, and neck [5]. After an extensive literature search, none of the cases was found to involve the spermatic cord. Here, we report a case of MPLPS from the spermatic cord which we managed in our institution.

## Case presentation

**Figure 1:**
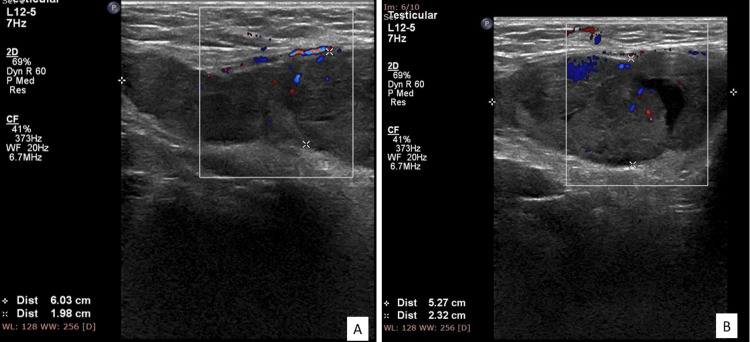
Ultrasound imaging of the scrotum Scrotal ultrasound showed a relatively well-defined hypoechoic lesion with internal cystic spaces measuring 6.0 x 1.9cm with areas of internal vascularity within the right inguinal canal region along the spermatic cord.

A 50-year-old male patient with no known co-morbid conditions, presented to the hospital with a swelling in the right inguinal region, which came to the notice of the patient in the past month. On clinical examination, a swelling of size 4x5 cm was noted in the right inguinal region, which was non-reducible, non-tender, and palpable separately from the right testis with no skin involvement. Both the left hemi-scrotum and the left inguinal area were normal. The testis on the left side with the cord structure seemed normal. Penis was positioned normally. The patient's general and systemic examination were normal. Preoperative ultrasound of the abdomen and scrotum was performed, which revealed a well-defined lobulated hypoechoic lesion with internal cystic spaces in the right inguinal canal along the spermatic cord showing internal vascularity with a differential of adenomatoid tumor/sarcomatous neoplasm of the spermatic cord was given. Bilateral testis and cord structures, along with the left inguinal region, were unremarkable. The Ultrasound sonographic features are shown in Figure 1. Considering the advanced nature of the disease, the patient was then planned for right-high inguinal orchidectomy, and the specimen was subjected to histopathological examination.

**Figure 2:**
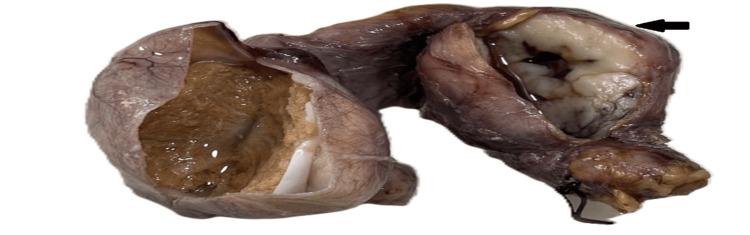
Gross image of the retrieved specimen Gross image of the right orchidectomy specimen with solid lobulated mass (black arrow) in spermatic cord

A gross examination (Figure 2) of the right orchidectomy specimen along with the cord showed an ovoid tumor of size 4x4x3.5 cm found within the cord. The cut surface of the tumor was solid, grey-yellow, and lobulated. The tumor was 2 cm away from the surgical resected margin of the cord. The right testis was grossly unremarkable.

**Figure 3:**
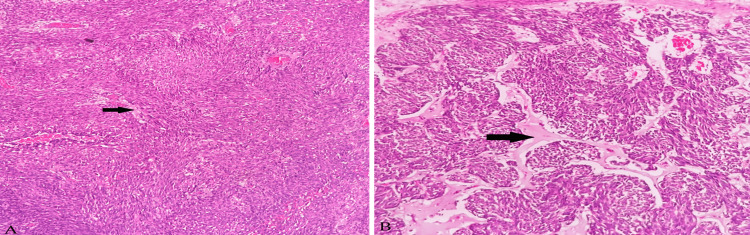
Microscopic examination image 1 Microscopic examination of the retrieved mass in low power view (H&E, 100X magnification) showing tumor cells arranged in fascicles and sheets forming lobules at places (A), along with intervening myxoid stroma (black arrows in A and B).

**Figure 4:**
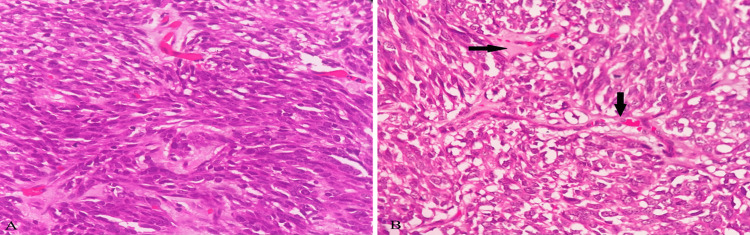
Microscopic examination image 2 Microscopic examination of the retrieved mass in high-power view (H&E, 400X magnification) showing spindled to ovoid-shaped cells with markedly pleomorphic elongated nuclei and increased mitotic activity along with cells with vacuolated cytoplasm and intervening myxoid stroma (A and B). Delicate arborizing blood vessels are shown in image B(black arrow).

**Figure 5:**
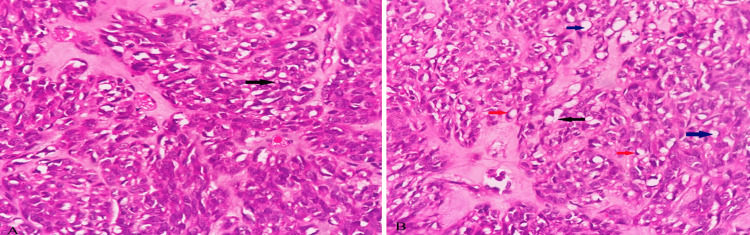
Microscopic examination image 3 Microscopic examination of the mass in high power view (H&E, 400X magnification) showing tumor cells with intervening myxoid stroma along with few lipoblasts (black arrows in images A and B), and adipocytes (blue arrows in image B). Also noted are a few cells with signet ring morphology (Red arrows in image B)

**Figure 6:**
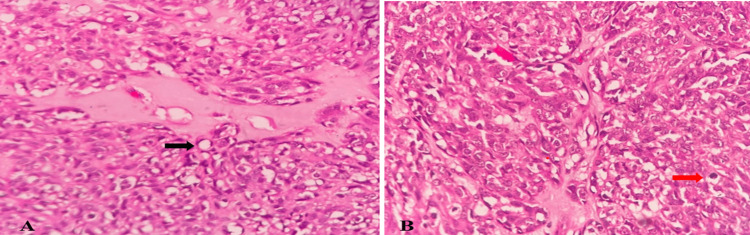
Microscopic examination image 4 Microscopic examination of mass in high power view (H&E, 400X magnification) showing cells with vacuolated cytoplasm having signet ring morphology (black arrow in image A) along with myxoid stroma. Increased mitotic activity shown (red arrow in image B) in myxoid stroma.

Microscopic examination of the tumor showed partially encapsulated, well-demarcated tumor cells arranged in fascicles, sheets, and forming lobules at places shown in Figure 3. The tumor cells are spindle to ovoid with moderate to markedly pleomorphic elongated nuclei with conspicuous nucleoli and high mitotic activity (>20/10 high power field). Other areas showed sheets of cells with polygonal morphology, round to ovoid nuclei exhibiting mild pleomorphism, and well-defined cell margins. A good number of cells showed vacuolated cytoplasm. In some places, cells showed adipocyte (focal signet ring cell) morphology with occasional lipoblasts. Intervening stroma showed myxoid changes. Few blood vessels were seen some of them showing perivascular myxoid changes and dense plasma cell aggregates. These histopathological findings are shown in Figures 4-6.

**Figure 7:**
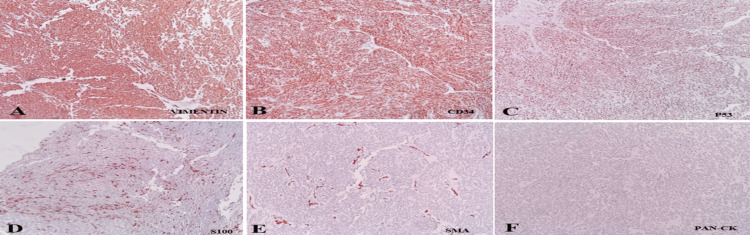
Immunohistochemistry Immunohistochemical analysis (100X magnification): A) Vimentin: strong membranous positivity in tumor cells; B) CD34: strong membranous positivity in tumor cells; C) p53: strong nuclear positivity in tumor cells; D) S100: focal positivity in tumor cells; E, F) SMA and PanCK: negative in tumor cells.

All these features were suggestive of liposarcoma with high-grade nuclear features with intervening myxoid stroma. For further subtype analysis, Immunohistochemistry was performed. p63 and PanCK were negative, and vimentin showed strong positivity in the tumor cells. Immunostaining with CD34, and P53 showed strong positivity along with focal positivity of S100 in the tumor cells. SMA was negative. Immunohistochemistry images are shown in Figure 7. Based on the histopathological and immunohistochemistry findings, the diagnosis of myxoid pleomorphic liposarcoma of the spermatic cord was made.

Considering the advanced nature of the disease, the patient initially underwent right-high inguinal orchidectomy. Intraoperatively no local extension of the disease was noted. Following histopathological confirmation of the diagnosis, the patient was then started on adjuvant chemotherapy. The patient was started on a doxorubicin-based chemotherapeutic regimen. A contrast-enhanced CT abdomen and positron emission tomography (PET) scan was done, which revealed no local or distant metastasis. Two months post procedure, no local recurrence was noted. Currently, the patient is doing well, and under follow-up.

## Discussion

Spermatic cord liposarcomas represent 3-7% of all paratesticular sarcomas, making them incredibly rare malignant tumors. The identification and treatment of them are challenging since they are clinically identical to testicular tumors [2]. It is typically in the fifth and sixth decades of life that these malignancies manifest. Often misdiagnosed as inguinal hernia, lipoma, hydrocoele, or spermatocele as these are painless, slow-growing masses in the inguinal or inguinoscrotal area [1,2,6].

According to the World Health Organization, liposarcomas can be classified as well-differentiated, de-differentiated, myxoid, pleomorphic, and myxoid-pleomorphic liposarcomas (MPLPS) [4]. MPLPS is a recently added subtype, recognized in 2009 by Alaggio et al. [7]. Among these, well-differentiated is the most common type, with approximately 40-50% of all liposarcomas whereas myxoid pleomorphic is an exceptionally rare variety with aggressive nature. This tumor has a high recurrence rate and can metastasize to lungs, bone, and soft tissues very early [4]. An aggressive nature was suggested by the 100% progression rate of these tumors in terms of either local recurrence or distant metastasis, as observed in a recent study involving eight cases. Of the 35 cases for which there was clinical follow-up available, 74% of them died within a few years of the diagnosis, according to the review [5]. It primarily affects children and adolescents, is incredibly rare, and prefers the mediastinum while it can occur in other places. In a recently published literature review, eight studies with a total of 39 cases were reviewed. The mean age was 29 years. Out of 39 cases, the mediastinum was the most common site involved, with a total of 22 cases, with six cases involving thighs, three cases involving the head, neck, and trunk, and a single case report involving leg and retroperitoneum. Recently, one case was reported for its rare location in the falciform ligament. None of the reviewed studies has shown the involvement of spermatic cord making our case a unique and rare entity [5,7-12].

MPLPS has been studied to be associated with Li-Fraumeni syndrome, with a pathogenic TP53 mutation [4]. In a recently published study, genetic screening was done for a patient with a strong family history and found its association with pathogenic TP53 gene mutation [13]. Our patient had no family history, so a genetic analysis was not done.

Histologically, myxoid liposarcomas consists of undifferentiated ovoid or spindle cells and small lipoblasts set in a copious myxoid matrix, with extremely scarce mitoses. The myxoid pleomorphic liposarcomas resemble conventional low-grade myxoid liposarcoma in places, but they also show areas of increased cellularity, marked nuclear pleomorphism, and large, bizarre lipoblasts with increased mitotic figures which were seen in our case [14].

Well-differentiated and de-differentiated varieties show MDM2 and CDK4 positivity, which helps differentiate them from other entities. CD34 and p16 will be negative in myxoid type, whereas these IHC markers will be positive in pleomorphic and myxoid pleomorphic varieties [15-17]. immunohistochemistry (IHC) analysis was done in a previous study and was found to be diffuse CD34 along with p16 expression, a loss of expression of nuclear Rb and MDM2 was negative [9]. Our study showed Vimentin positivity favoring mesenchymal origin and, CD34 and P53 positivity consistent with the available literature, favoring the diagnosis of MPLPS.

Fluorescence in situ hybridization (FISH) analysis was done in some of the studies before, which revealed FUS/EWSR1-DDIT3 fusion to be negative and also the absence of MDM2 amplification in this type, which differentiates MPLPS from the conventional myxoid liposarcoma [7,9]. According to WHO, FISH analysis is the only desirable criterion that can be used in selected cases where the essential histopathological criteria become inconclusive. As the essential criteria of admixed features of both conventional myxoid liposarcoma and pleomorphic type were present in our case, along with the convincing evidence of MPLPS by IHC, FISH analysis was not done in our case.

Treatment for this condition is always surgical intervention, which is the prime choice. Due to the rarity of the condition, none of the treatment protocols have been devised particularly for individual subtypes. To improve local control and lower the risk of distant metastasis, treatment should aim for a thorough resection of the tumor with broad margins, followed by adjuvant radiotherapy [18]. Chemotherapy regimens are available for soft tissue sarcomas like AIM (doxorubicin, ifosfamide, mesna), gemcitabine, and docetaxel [19,20]. Adjuvant chemotherapy was tried in a study that used these regimens. Despite aggressive management, all the patients experienced rapid disease progression [4]. In our case, due to the aggressive nature, we performed a right-sided high orchidectomy followed by adjuvant chemotherapy to prevent recurrence and early metastasis. As the tumor mostly involves children and young adults, individualized treatment protocols need to be devised considering the aggressive nature of this subtype.

## Conclusions

With relatively few occurrences documented in the literature, myxoid pleomorphic liposarcoma (MPLPS) is an incredibly rare entity. With the most common site being mediastinum, ours is the first case of MPLPS presenting in the spermatic cord. This entity must be considered by clinicopathologists because of the aggressive nature of the disease for early diagnosis and treatment strategies.
